# Can apps be used to formulate fluid therapy plans in veterinary medicine?

**DOI:** 10.1111/jvim.16526

**Published:** 2022-09-22

**Authors:** Simona Aukse Zduoba, John House, Sam Rowe

**Affiliations:** ^1^ Sydney School of Veterinary Science The University of Sydney Camden New South Wales Australia

**Keywords:** app, diarrhea, fluid therapy, veterinary medicine

## Abstract

**Background:**

Formulating sophisticated fluid therapy plans can be complicated and time consuming. Consequently, veterinarians in the field who lack experience, time, or confidence may formulate suboptimal fluid therapy plans.

**Objectives:**

Compare conventional and app‐guided fluid therapy plans for simulated cases of neonatal calf diarrhea.

**Participants:**

Third and fourth year veterinary students (n = 55) from The University of Sydney.

**Methods:**

We developed a web app to assist fluid therapy formulation (http://calfaid.com) that was evaluated in a randomized case simulation trial. Participants were instructed to perform fluid therapy calculations and formulate an integrated fluid therapy plan for case scenarios using conventional methods and using the fluid therapy app. Responses were scored by a blinded study investigator using an a priori scoring guide and groups (conventional vs. app‐guided) were compared using linear mixed models.

**Results:**

On average, total scores for app‐guided fluid therapy calculations were 20.6% points higher (95% confidence interval [CI], 14.1‐27.1) than calculations completed using the conventional method (88.2% vs. 67.5%, respectively). On average, total scores for app‐guided integrated fluid therapy plans were 14.2% points higher (95% CI, 6.3‐22.2; 65.8% vs. 51.2%). Eighty percent of respondents indicated they would prefer to use the app‐guided method over the conventional method.

**Conclusion and Clinical Importance:**

Our findings suggest that fluid therapy plans can be improved using apps.

## 1 INTRODUCTION

Diarrhea is a common disease of neonates in many species. In many cases, affected animals can develop dehydration, metabolic acidosis, and electrolyte imbalances, which can be corrected with fluid therapy. However, the beneficial effects of fluid therapy require that the fluid plan be appropriately designed. For example, a recent study^1^ showed that administering suboptimal volumes of IV isotonic fluids to dehydrated calves was associated with delayed recovery. In cattle, diarrhea is the most common cause of preweaning morbidity and mortality.^2^ Designing an appropriate and effective fluid therapy plan for calves requires numerous assessments of the patient, including measurement of body weight, fluid deficit, maintenance requirements, ongoing losses, and base deficit. Given that most calves with diarrhea are treated on farms, direct measurement of critical variables is rarely possible and expected results usually are estimated usi “rules of thumb.” For example, blood gas analysis is rarely available to directly measure blood pH and serum bicarbonate concentrations, and consequently, multivariable algorithms have been derived to estimate base deficit, based on calf age, ability to stand, suck reflex, and palpebral reflex.^3^, ^4^, ^5^

After patient assessment, numerous additional calculations are required to design a fluid therapy plan that includes appropriate fluid types, routes and rates of administration, and additives to facilitate rapid recovery. Consequently, designing an appropriate fluid therapy plan can be challenging and time‐consuming for clinicians with limited experience, and in some situations may lead to the adoption of generic (i.e., nontailored) fluid plans or fluid plans based on erroneous calculations. To simplify the process, treatment guides (flowcharts and tables) have been developed and are found in most veterinary medicine textbooks.^5^, ^6^, ^7^ However, these treatment guides fail to incorporate total fluid requirements, do not provide the user with flexibility to choose a specific fluid type, and do not provide detailed guidance for rates of administration. In summary, tools that simplify the development of fluid therapy plans are needed in veterinary medicine.

Smart phone apps have been developed and validated to assist calculations in clinical environments in human medicine. For example, a recent clinical trial found that Swiss paramedics were 66.5% less likely to make medication errors when using an app to calculate doses of epinephrine, midazolam, 10% dextrose, and sodium bicarbonate for a simulated cardiopulmonary resuscitation scenario for an 18‐month‐old child.^8^ We hypothesized that a fluid therapy app would enable veterinarians to efficiently formulate fluid therapy plans that are more consistent with best practice. However, it is currently unclear if this tool, and other tools like it, would be sufficient to meet the needs of veterinarians in practice. Our objective was to compare conventional and app‐guided fluid therapy plans for calves with diarrhea in a randomized case simulation trial.

## 2 MATERIALS AND METHODS

A randomized trial was conducted using an online questionnaire to compare fluid therapy plans formulated using conventional and app‐guided methods. All procedures in the study were conducted in accordance with The University of Sydney Research Ethics Committee (Protocol # 2021/140). Informed consent was obtained from the participants and the questionnaire was voluntary, anonymous, and untimed. It was originally estimated that 50 students would be necessary to demonstrate a 20% point difference in scores for app‐guided (80%) and conventional (60%) fluid therapy plans, assuming a SD of 30%, alpha of 0.05, and power of 0.80.

### 2.1 Enrollment of participants

Participants were veterinary students at The University of Sydney in 2021. Students in their fourth year of study (DVM4, n = 110) were invited to participate during their livestock medicine rotation (March 22, 2021 to October 5, 2021) and the third year students (DVM3, n = 120) were invited on August 2, 2021 after having completed lectures and examinations in the topic of large animal fluid therapy. The online questionnaire was closed on October 5, 2021. At the time of the study, veterinary students from both cohorts had 1 lecture that was focused on livestock fluid therapy in third year where they learn about fluid types and routes and rates of administration. Students were also taught a systematic approach to performing fluid therapy for calves, including estimation of clinical variables (e.g., % dehydration, base deficit) and a guide to performing calculations (e.g., fluid deficit, ongoing losses, maintenance). During the clinical rotation in the fourth year, students did not receive structured learning activities in the topic of fluid therapy, but participated in treating clinical cases that presented to the Livestock Veterinary Services at The University of Sydney. The livestock courses and clinical rotations were compulsory for all students, because “tracking” in a specific discipline of veterinary medicine is not available in veterinary schools in Australia.

### 2.2 Development of the fluid therapy app

**FIGURE 1:**
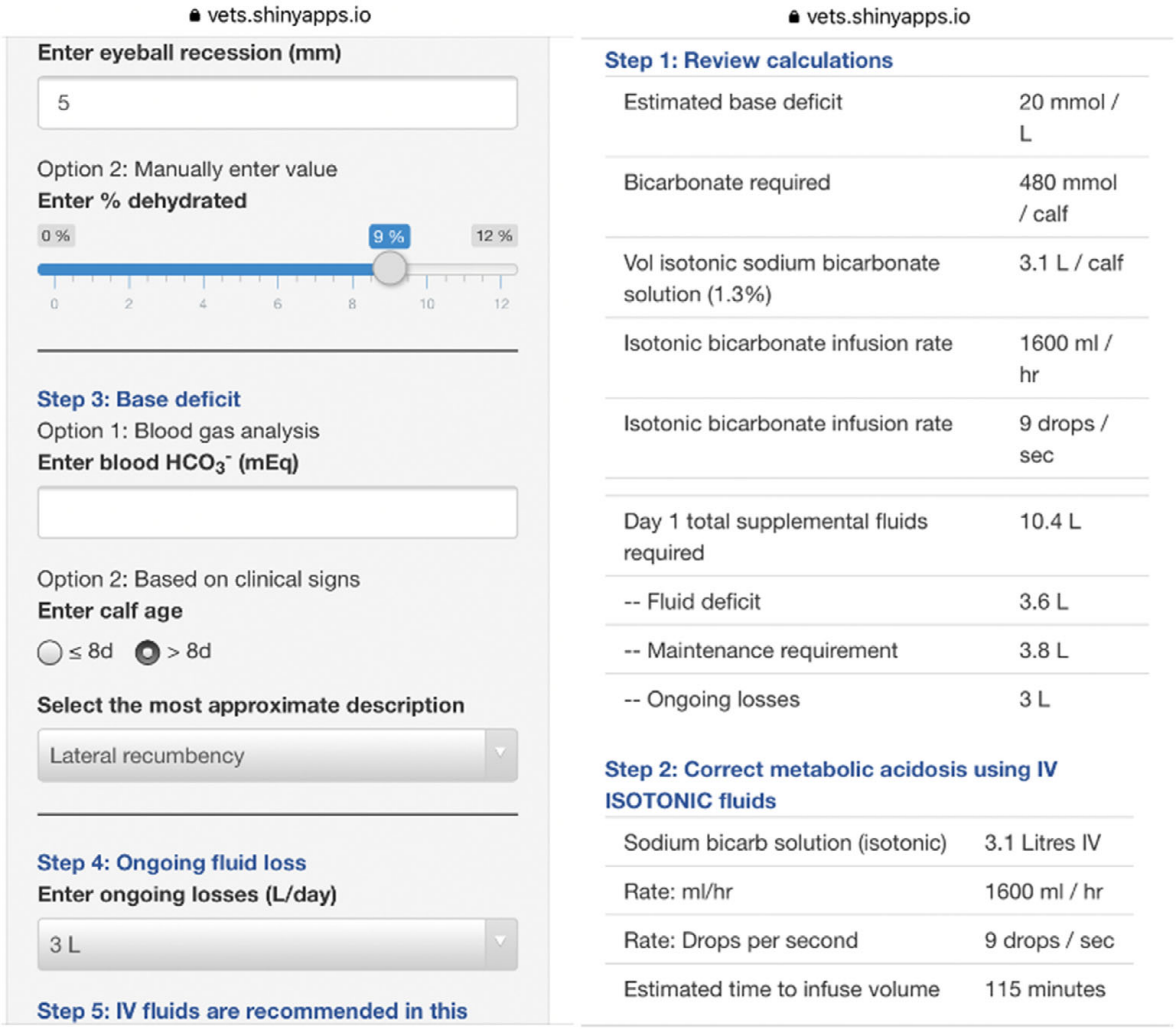
Screenshots of an app used to create fluid therapy for calves with diarrhea. The app can be viewed at: http://calfaid.com

A Shiny web app that helps users formulate fluid therapy plans for dehydrated calves (http://calfaid.com) was developed by our group in R.^9^ It is a free web app that can be used on any device with a web browser (e.g., smartphone, tablet, or computer). The app was designed after reviewing peer‐reviewed clinical research, review articles and large animal medicine textbooks. At the time of the study, users could determine dehydration based on the severity of enophthalmos (% dehydrated = eyeball recession in mm × 1.7),^10^ maintenance requirement for neonatal calves was set at 4 mL/kg/h and users were able to set ongoing losses at values of 1 to 4 L per day.^4^, ^5^, ^6^, ^11^, ^12^ Acid‐base imbalances were estimated using direct measurements of bicarbonate concentration in blood or by using a previously described algorithm,^5^ which is based on ability to stand, suck reflex, and age. We decided to use the previously proposed method^5^ instead of a more recently validated method,^3^, ^13^ because it was the method routinely used in our veterinary clinic. Users had the options to correct base deficits with isotonic (1.3%) or hypertonic (8.4%) bicarbonate solutions, with maximum rates of administration set at 80 mL/kg/h^14^ and 60 mL/kg/h (equivalent to 1 mL/kg/min),^12^ respectively. In addition, users could estimate body weight using heart girth, based on a previously developed formula.^15^ After clinical information was entered into the app, it generated a review table that summarized the estimated base deficit (mmol/L), bicarbonate requirement (mmol/calf), volume of sodium bicarbonate solution required (L/calf), infusion rate (mL/h and drops/second), and 24‐hour fluid requirements (i.e., fluid deficit, maintenance, ongoing losses). Afterward, the app outlined the steps required to correct metabolic acidosis, correct the remaining fluid deficit after correction of metabolic acidosis, and PO fluid requirements to account for maintenance and ongoing losses. Screenshots of the app are presented in Figure 1.

### 2.3 Study design

An online questionnaire was administered using Qualtrics software. A PDF version of the survey can be downloaded from Data S1, Supporting Information. Demographic information collected about participants included: age, sex, year of study (DVM3 or DVM4), and if they used smart phone apps as part of their study routines. Participants were provided with 2 hypothetical case scenarios. Case A described a 3‐week‐old calf that weighed 40 kg, had profuse watery diarrhea, sunken eyes (4 mm), impaired mentation, absent suck reflex, inability to stand, and normal blood glucose concentration. Case B described a 5‐day‐old calf that weighed 30 kg, had profuse watery diarrhea, sunken eyes (5 mm), imp mentation, absent suck reflex, inability to stand, and normal blood glucose concentration. The questionnaire was randomized so that approximately half of the participants would complete Case A before Case B, and half of the participants would complete Case B before Case A. For each case, participants were prompted to calculate the fluid deficit, maintenance requirements, ongoing losses, and to estimate the base deficit (these 4 calculations are hereafter referred to as “fluid therapy calculations”). In addition, participants were instructed to formulate a fluid therapy plan for the first 24 hours for each case, including the fluid type or types, volume, rate of administration and additives required (hereafter referred to the “integrated fluid plan”).

**FIGURE 2:**
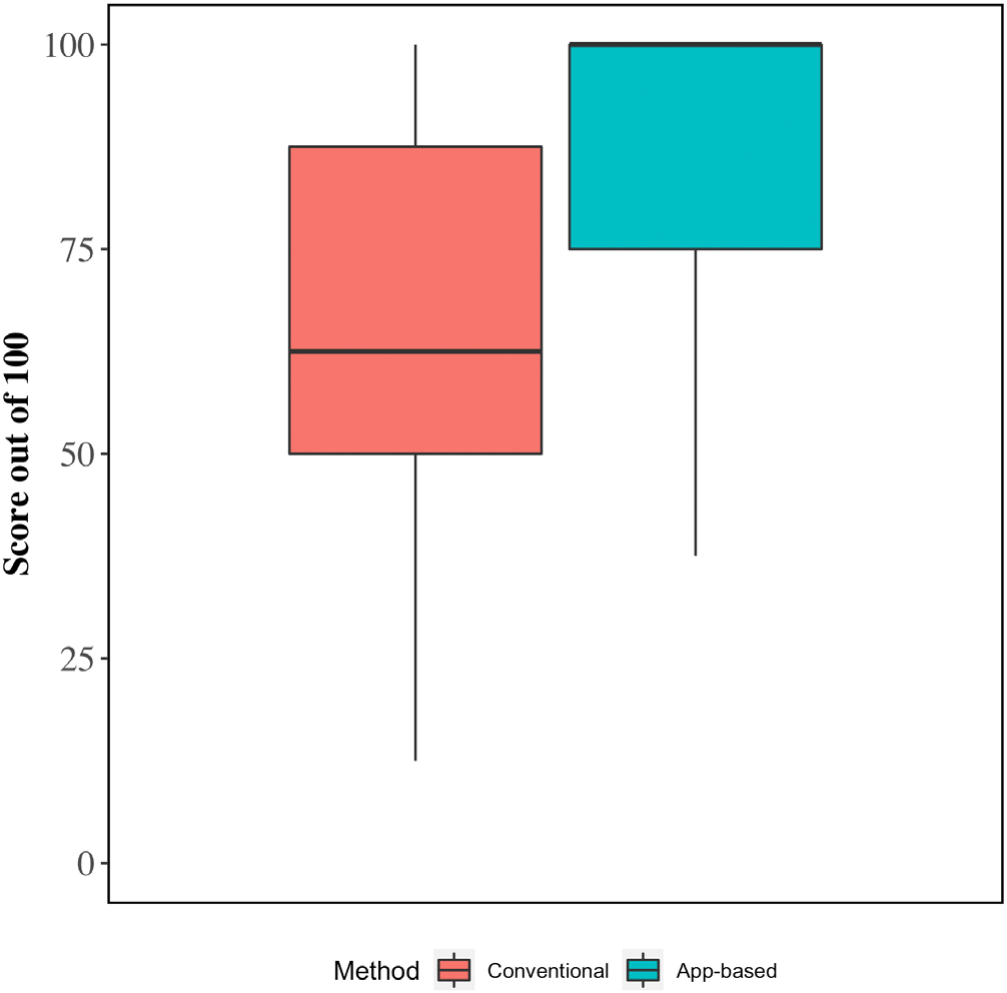
Boxplots showing 25th, 50th (median), and 75th percentiles of scores (out of 100) for fluid therapy calculations using conventional and app‐guided methods. The score is based on the accuracy of calculations for fluid deficits, maintenance requirements, ongoing losses, and base deficits (see Table 1). Lower and upper whiskers extend from the 25th and 75th percentiles to the furthest data point that is within 1.5× the interquartile range distance from the 25th and 75th percentiles, respectively

For the first case (A or B), participants were instructed to perform calculations and to formulate their integrated fluid plan using a “conventional” approach, which included the use of lecture material and textbooks. To facilitate doing so, students were provided with a link to download the lecture slides from their DVM curriculum that provided instructions on formulating fluid therapy plans for calves with diarrhea. For the second case (A or B), students were provided a link to the fluid therapy app and instructed to use it to complete the same tasks that were done for the previous case. No instructions were provided on how to use the app. Case scenarios were designed such that students had the sufficient information in the lecture notes and in the app to score 100% for all questions (Figure 2).

After the completion of both cases, participants were asked the following questions with Likert scale responses: “Describe your level of confidence in the accuracy of your calculations for the case that was completed using the conventional method” and “How user‐friendly was the interface for the app?” Students also were asked: “If you were expected to design a fluid therapy plan for a sick calf in the field, which method would be your preference?”

### 2.4 Data management and scoring of responses

The responses from the survey were collated and recorded in Qualtrics and imported into Microsoft Excel (Redmond, Washington) at the end of the study. Participants who did not attempt to answer the questions relating to the case scenarios were excluded from analysis. Scoring guides were created to determine the accuracy of fluid calculations and integrated fluid plans. The investigator who scored responses was blinded to the method used by the participant (ie, conventional vs. app‐guided). Scoring of fluid therapy calculations evaluated the participants' ability to estimate fluid deficits, ongoing losses, maintenance requirements and base deficits. Each component was scored out of a total of 2, with a value of 2 being assigned to responses that were within an a priori range that we deemed to be acceptable, based on our subjective assessment for how effective the fluid therapy plan would be based on calculated outcomes. A value of 1 was given if the response was outside of the acceptable range, but close enough to demonstrate some accuracy in the calculation. A score of 0 was given if the response was outside of the ranges for scores 1 or 2. All ranges were established before data analysis was conducted and are shown in Table 1. For example, in Case A, the hypothetical calf was estimated to have a fluid deficit of 2.7 L according to the “rules of thumb” used in our clinic: 4 mm eyeball recession × 1.7/100 × 40 kg live weight. To score 2 points for this component, participants had to record a value between 2.5 and 2.9 L (i.e., within ±0.2 of 2.7 L). To score 1 point, participants had to record a value between 1.9 and 3.5 L (i.e., within ±0.8 of 2.7 L) and not between 2.5 and 2.9 L.

**TABLE 1:** Scoring guide for fluid therapy calculations

	Case A Age: 3 weeks Body weight: 40 kg Clinical signs: sunken eyes (4 mm), profuse watery diarrhea, lethargic mentation, absent suck reflex, inability to stand Blood glucose: normal			Case B Age: 5 days Body weight: 30 kg Clinical signs: sunken eyes (5 mm), profuse watery diarrhea, lethargic mentation, absent suck reflex, inability to stand Blood glucose: normal		
	Correct	2 points	1 point	Correct	2 points	1 point
% dehydrated	6.8	6.3‐7.3	4.8‐8.8	8.5	8‐9%	7‐10%
Fluid deficit (L)	2.7	2.5‐2.9	1.9‐3.5	2.6	2.4‐2.8	2.1‐3.0
Ongoing losses (L)	3‐4	3‐4	2‐5	3‐4	3‐4	2‐5
Maintenance (L)	3.8	2.9‐4.8	2.4‐5.3	2.9	2.5‐3.2	1.8‐4.0
Base deficit (mmol/L)	15‐20	15‐20	10‐20	10	10	5‐15

Scoring of responses for the integrated fluid therapy plan was done in a similar way to scoring of fluid calculations, except that each component (n = 6) was assigned a score of 0 or 1 (Table 2). The 6 components in the scoring guide included: “Was the total fluid volume sufficient to rehydrate the patient?” (ie, Did the total volume prescribed exceed the fluid deficit?), “Was the total fluid volume appropriate to cover the fluid requirements in the first 24 hours? (i.e., did the total fluid volume meet the requirements for fluid deficit, maintenance and ongoing losses?), “Did the fluid plan include the use of an alkalinizing solution?,” “Was the volume of the alkalinizing solution appropriate?,” “Was the rate of administration of the alkalinizing solution appropriate?,” and “Did the plan include the use of oral fluids?” Components that were within an a priori range deemed acceptable by the investigators were assigned scores of 1 with all other responses assigned scores of 0. All ranges are shown in Table 2.

**TABLE 2:** Scoring guide for evaluating the integrated fluid therapy plan

	Case A Age: 3 weeks Body weight: 40 kg Clinical signs: sunken eyes (4 mm), profuse watery diarrhea, lethargic mentation, absent suck reflex, inability to stand Blood glucose concentration: normal		Case B Age: 5 days Body weight: 30 kg Clinical signs: sunken eyes (5 mm), profuse watery diarrhea, lethargic mentation, absent suck reflex, inability to stand Blood glucose concentration: normal	
	Score 0	Score 1	Score 0	Score 1
Was the total fluid volume sufficient to rehydrate the patient?	Total volume of fluid does not exceed the fluid deficit volume for this case (2.7 L)	Total volume of fluid exceeds the fluid deficit volume for this case (2.7 L)	Total volume of fluid does not exceed the fluid deficit volume for this case (2.6 L)	Total volume of fluid exceeds the fluid deficit volume for this case (2.6 L)
Was the total fluid volume appropriate to cover the fluid requirements in the first 24 hours?	Total volume of fluid in the first 24 hours is not between 8 and 10 L	Total volume of fluid in the first 24 hours is between 8 and 10 L	Total volume of fluid in the first 24 hours is not between 7 and 9 L	Total volume of fluid in the first 24 hours is between 7 and 9 L
Did the fluid plan include the use of an alkalinizing solution?	Plan does not include any alkalinizing solution	Plan mentions alkalinizing solution	Plan does not include any alkalinizing solution	Plan mentions alkalinizing solution
Was the volume of the alkalinizing solution appropriate?^a^	Plan does not mention any alkalinizing solution or value outside of range (2.0‐3.1 L)	Answer mentions an alkalinizing solution of 2‐3.1 L	Plan does not mention any alkalinizing solution or value outside of range (0.9‐1.5 L)	Answer mentions an alkalinizing solution of 0.9‐1.5 L
Was the rate of administration of the alkalinizing solution appropriate?^a^	Answer does not mention a range of 20‐80 mL/kg/h for bicarbonate OR 0.8‐3.2 L/h OR 4‐18 drops per second	Answer should mention a range of 20‐80 mL/kg/h for bicarbonate OR 0.8‐3.2 L/h OR 4‐18 drops per second	Answer does not mention a range of 20‐80 mL/kg/h for bicarbonate OR 0.6‐2.4 L/h OR 3‐13 drops per second	Answer should mention a range of 20‐80 mL/kg/h for bicarbonate OR 0.6‐2.4 L/h OR 3‐13 drops per second
Did the plan include the use of oral fluids?	Plan did not mention any oral solutions	Answer includes the use of oral solutions such as milk, oral electrolyte solutions, or both	Plan did not mention any oral solutions	Answer includes the use of oral solutions such as milk, oral electrolyte solutions, or both

^a^ Volumes and rates for isotonic bicarbonate solution (1.3%) are shown in this scoring guide as all students chose to use this type of alkalinizing solution (ie, this is why no criteria are not shown for 5% of 8.4% solutions of bicarbonate).

### 2.5 Outcomes

The 2 major quantitative outcomes in our study were (a) total score for fluid therapy calculations (sum of the 4 components) and (b) total score for integrated fluid therapy plan (sum of 6 components). Total scores were expressed as percentages. For example, a response with the following component scores for fluid deficit (1/2), ongoing losses (2/2), maintenance (2/2), and base deficit (2/2) would have a total score for fluid therapy calculations of 87.5% (7 out of 8).

### 2.6 Statistical analysis

Analysis was conducted in R.^9^ Total scores for app‐guided and conventional methods were compared for fluid therapy calculations (outcome 1) and integrated fluid therapy plans (outcome 2) using linear mixed models in the lme4 package.^16^ Models included fixed effects for fluid therapy method (conventional vs. app‐guided) and case type (5‐day vs. 3‐week old calf scenarios). A random intercept for each participant was used to account for the clustering of responses within participants. For both outcomes, the betacoefficient for the method term indicated the average difference in total scores (app‐guided minus conventional method). The crude differences in individual components that contributed to total scores (eg, ongoing losses calculation) were calculated using the crude means. No models were conducted to estimate the differences in scores for individual components because the data were unlikely to be normally distributed and thus not appropriate for linear regression models. A complete analysis log, which includes the dataset can be accessed at https://rpubs.com/samrowe/fluid_app_2022.

## 3 RESULTS

### 3.1 Description of participants

Fifty‐eight questionnaire responses were returned between March 22 and October 5, 2021, of which 55 were eligible for inclusion into the final dataset. On average, participants were 26.7 years of age at the time of enrollment (SD 3.8; median, 26; range 22‐41), 37 (67.3%) were female, and 18 (32.7%) were male. Twenty‐one (38.2%) students were in the third year of their veterinary degree, and 34 (61.8%) were in their fourth year. The response rates from DVM3 and DVM4 students were 17.5% (21/120) and 30.9% (34/110), respectively. Thirty‐three (60%) students indicated that they used apps as part of their study routine. The number of participants who were randomized to complete Case A using the conventional method and Case B using the app‐guided method was 30 (55.5%). The remaining 25 (45.5%) participants completed Case B using the conventional method before completing Case A using the app‐guided method.

### 3.2 Comparison of conventional and app‐guided fluid plans

#### 3.2.1 Fluid therapy calculations

Crude average scores for fluid therapy calculations and model‐estimated differences between app‐guided and conventional methods are shown in Table 3 and Figure 2. On average, total scores for app‐guided fluid therapy calculations were 20.6% points higher (95% confidence interval [CI], 14.1‐27.1) than calculations completed using the conventional method (88.2% vs. 67.5%, respectively). The higher level of overall accuracy was the result of higher scores when calculating fluid deficits (+25.5% points), ongoing losses (+20.9%), maintenance requirements (+17.3%), and base deficits (+19.1%).

### 3.3 Integrated fluid therapy plans

**FIGURE 3:**
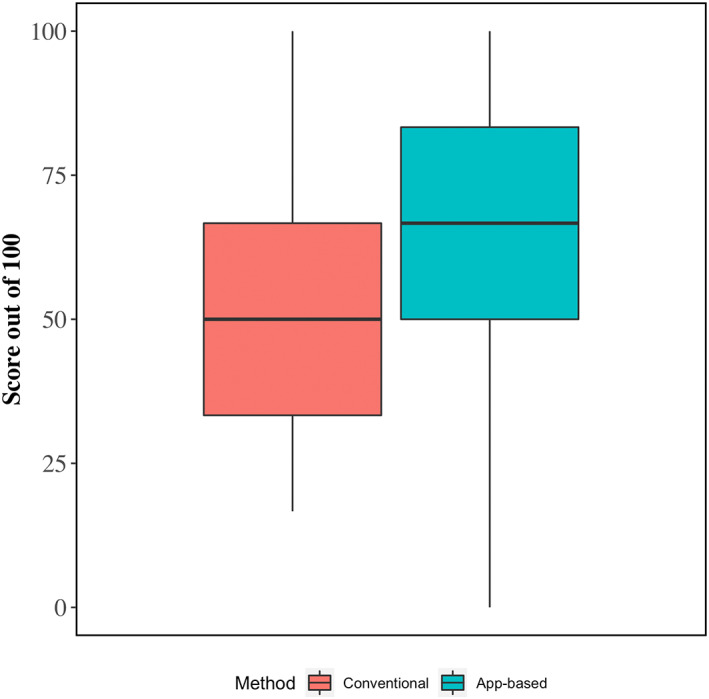
Boxplots showing 25th, 50th (median), and 75th percentiles of scores (out of 100) for integrated fluid therapy plans using conventional and app‐guided methods. Fluid therapy plans were evaluated according to a scoring guide that identified if the plans were sufficient to replace fluid deficits, meet the total 24 hours fluid requirement, use of an alkalinizing solution to correct metabolic acidosis, and use of oral fluids (see Table 2). Lower and upper whiskers extend from the 25th and 75th percentiles to the furthest data point that is within 1.5× the interquartile range distance from the 25th and 75th percentiles, respectively

Crude average scores for integrated fluid therapy plans and model‐estimated differences between app‐guided and conventional methods are shown in Table 3 and Figure 3. On average, total scores for app‐guided integrated fluid therapy plans were 14.2% points higher (95% CI, 6.3‐22.2) than fluid therapy plans formulated using the conventional method (65.8% vs. 51.2%, respectively). The higher overall score was a result of app‐guided fluid therapy plans having more appropriate volumes of alkalinizing agents (+27.3%), more appropriate rates of administration for alkalinizing agents (+25.5%), and the inclusion of oral fluids (+38.2%).

**TABLE 3:** Comparison of calculations and integrated fluid therapy plans when using a conventional and app‐guided method

	Average score (%)		Difference (95% CI)
	Conventional	App	
Fluid calculations			
Total score (model 1)	67.5	88.2	+20.6 (14.1, 27.1)^a^
Deficit	61.8	87.3	+25.5
Ongoing losses	60.0	80.9	+20.9
Maintenance	77.3	94.5	+17.3
Base deficit	70.9	90.0	+19.1
Integrated fluid therapy plan			
Total score (model 2)	51.2	65.8	+14.22 (6.25, 22.19)^a^
Will the plan rehydrate the patient?	90.9	87.3	−3.6
Will the plan cover 24 hour fluid requirements?	34.5	30.9	−3.6
Is an alkalinizing agent included?	87.3	90.9	+3.6
Is the alkalinizing agent volume appropriate?	36.4	63.6	+27.3
Is the alkalinizing agent rate appropriate?	50.9	76.4	+25.5
Are oral fluids (electrolyte +/− milk) included?	7.3	45.5	+38.2

^a^ Differences and their 95% confidence intervals (CI) were determined for total scores using linear mixed models. Differences reported for individual components (ie, those without 95% CI) are crude differences.

### 3.4 User experiences and preferences for formulating fluid therapy plans

When asked “Describe your level of confidence in the accuracy of your calculations” for the case that was completed using the conventional method, participants responded with very high (0%), high (3.8%), moderate (45.3%), low (39.6%), and very low (11.3%). When asked “How user friendly was the interface for the app?,” the responses were very easy to use (34.5%), easy to use (49.1%), neutral (10.9%), hard to use (1.8%), and very hard to use (0%). When asked “If you were expected to design a fluid therapy plan for a sick calf in the field, which method would be your preference?,” 80.0% of respondents indicated they would prefer to use the app‐guided method, 10.9% indicated that they would use a combination of the app‐guided and conventional methods, 3.6% indicated that they would prefer to use the conventional method, and 5.5% of students did not respond.

## 4 DISCUSSION

### 4.1 Use of an app could improve fluid therapy plans in the field

In this randomized trial we found that the use of an app improved the accuracy of fluid therapy calculations and enabled veterinary students to formulate integrated fluid plans that were more closely aligned with best practice, as evidenced by higher scores for fluid therapy calculations (+20.6%; 95% CI, 14.1‐27.1) and integrated fluid therapy plans (14.2; 95% CI, 6.3‐22.2). We are not aware of other studies that have quantitatively evaluated the use of apps to guide fluid therapy in veterinary medicine. However, findings from studies in human medicine are consistent with ours. For example, a recent clinical trial^8^ found that Swiss paramedics were 66.5% less likely to make medication errors when using an app to calculate doses of medication required to perform cardiopulmonary resuscitation in a hypothetical 18‐month‐old child. Likewise, a study of 28 pediatricians and 7 medical students conducted in Northern Ireland found that using a drug dosing app enabled 100% of participants to correctly prescribe inotropic infusions for simulated cases, whereas only 28.6% of participants could do so without using the app. Furthermore, the authors found users could perform calculations 5 minutes and 17 seconds faster on average when using the app.

In our study, the higher score for fluid therapy calculations resulted from higher accuracy when estimating fluid deficits (+25.5%), ongoing losses (+20.9%), maintenance requirements (+17.3%), and base deficits (+19.1%; Figure 3). These findings indicate that in this context, no specific calculation was markedly more difficult to perform than others, and that use of the fluid therapy app may be useful for all calculations. In contrast, when evaluating integrated fluid therapy plans, only certain components scored higher when using the app, including alkalinizing solution volume (+27.3%), alkalinizing solution rate of administration (+25.5%), and use of oral fluids (+38.2%) (Table 3). We believe that the markedly higher scores for alkalizing solution administration is a particularly important feature of the app, because rapid correction of diarrhea‐associated metabolic acidosis is critically important in human and animal patients.^17^ However, supplementation of bicarbonate is a relatively complicated component of fluid therapy plans, because it requires accurate estimation of the base deficit of the animal, as well as consideration of the molecular weight of sodium bicarbonate and concentration of sodium bicarbonate in isotonic or hypertonic solutions. This propensity for errors when supplementing sodium bicarbonate solutions is well recognized and is likely why most large animal medicine textbooks include tables to simplify this calculation.^5^, ^6^, ^7^ In the aforementioned study of Swiss paramedics, study investigators found that 100% of calculations for sodium bicarbonate infusions included medication errors, whereas only 6.8% of errors occurred when using the app‐guided method for the same calculation.^8^ In summary, the improved scores for app‐guided fluid plans in our study indicate that outcomes for calves with diarrhea that require IV fluid therapy potentially could be improved by use of an app‐guided method. However, intervention studies involving real cases (i.e., not case scenarios) and practicing veterinarians in the field are needed to definitively demonstrate this supposition. Furthermore, the effectiveness of an app‐guided fluid therapy plan will depend on the accuracy of the methods used to estimate critical variables. For example, the app uses heart girth measurements to estimate body weight, ocular recession to estimate dehydration, and clinical signs to estimate base deficit, which are not perfect substitutes for direct measurement. Therefore, users should measure these variables directly when it is possible to do so.

### 4.2 The use of apps in veterinary medicine is likely to increase

We believe that apps will be increasingly used to guide therapy in veterinary medicine in the future. This expectation is supported by our findings. For example, we found that 60.0% of participants in our study already used smart phone apps in their veterinary learning. Furthermore, 80.0% of participants indicated that they preferred the app‐guided method for developing fluid therapy plans and 83.6% indicated that the app was either “very easy” or “easy” to use despite not having been shown how to use the app at any point in the study. Peer‐reviewed articles have documented the use of apps in veterinary medicine in small animals,^18^ horses,^19^ food animals,^20^ and exotic animals.^21^ Furthermore, smart phone apps are ubiquitous in human medical disciplines, such as burn management,^22^ dermatology,^23^ pediatrics,^24^ emergency medicine,^25^ and surgery.^26^

### 4.3 Study strengths and limitations

We encourage readers to consider the strengths and limitations of our study when drawing conclusions from the findings. Some important strengths include the use of blinding when scoring fluid therapy plans, the use of an a priori scoring guide, and the use of randomization for allocating the order of cases to participants. Several limitations also should be considered. For example, all participants used the conventional method before the app‐guided method. It is possible that completing the first case with the conventional method may have served as practice for the next case and therefore artifactually improved app‐guided fluid therapy plans. However, we believe that this carry‐over effect is unlikely to be clinically relevant, because the app was designed to eliminate calculations that would have been practiced in the first case. It was not possible to randomize half of the participants to complete the first case using the app‐guided method (ie, a true “cross‐over” design) because we could not restrict access to the app once it was provided. It is also important to acknowledge the limitations of using an online questionnaire of simulated cases that was completed by third and fourth year veterinary students as a proxy for fluid therapy plans conducted in the field. First, it is unknown how representative veterinary students are of practicing cattle veterinarians, because veterinary students have considerably less experience. Second, it is possible that written responses to case simulations may differ from how participants would respond if faced with a real case in the field, because it lacks the practical factors that often are present in the clinical context, such as limited time and intrinsic and extrinsic motivations to help the patient and client. Finally, the a priori scoring scheme that we used is likely to be an imperfect quantitative measure of fluid therapy efficacy in the field. For example, it cannot be assumed that a fluid therapy plan that has a score 2 times higher than another plan is going to be 2 times more likely to prevent death in a patient.

### 4.4 Future research

Field‐based intervention studies should be conducted with practicing veterinarians to see if app‐guided fluid therapy can improve health outcomes and decrease time spent formulating fluid plans. Furthermore, we believe that fluid therapy apps could be developed to incorporate machine learning algorithms to predict base deficits from clinical signs.

## 5 CONCLUSION

In our randomized trial, we found that app‐guided fluid therapy plans were more closely aligned with best practice, as evidenced by higher scores for fluid therapy calculations and integrated fluid therapy plans. Therefore, additional studies are needed to determine if outcomes for calves with diarrhea can be improved using an app‐guided method.

## CONFLICT OF INTEREST DECLARATION

The authors developed the app evaluated in this study, but have not, and will not, benefit financially from its use.

## OFF‐LABEL ANTIMICROBIAL DECLARATION

Authors declare no off‐label use of antimicrobials.

## INSTITUTIONAL ANIMAL CARE AND USE COMMITTEE (IACUC) OR OTHER APPROVAL DECLARATION

Approved by the University of Sydney Human Research Ethics Committee (Protocol # 2021/140).

## HUMAN ETHICS APPROVAL DECLARATION

Authors declare human ethics approval was not needed for this study.

## Supporting information


**Data S1** Supporting Information.
