# Supplementary material for: Can apps be used to formulate fluid therapy plans in veterinary medicine?
Source: J Vet Intern Med. 2022 Sep 22;36(6):2079–87. doi: 10.1111/jvim.16526 (PMC9708415; doi:10.1111/jvim.16526)
Supplement: Supplementary file 1 — Data S1 Supporting Information. [file JVIM-36-2079-s001.pdf]

Intro

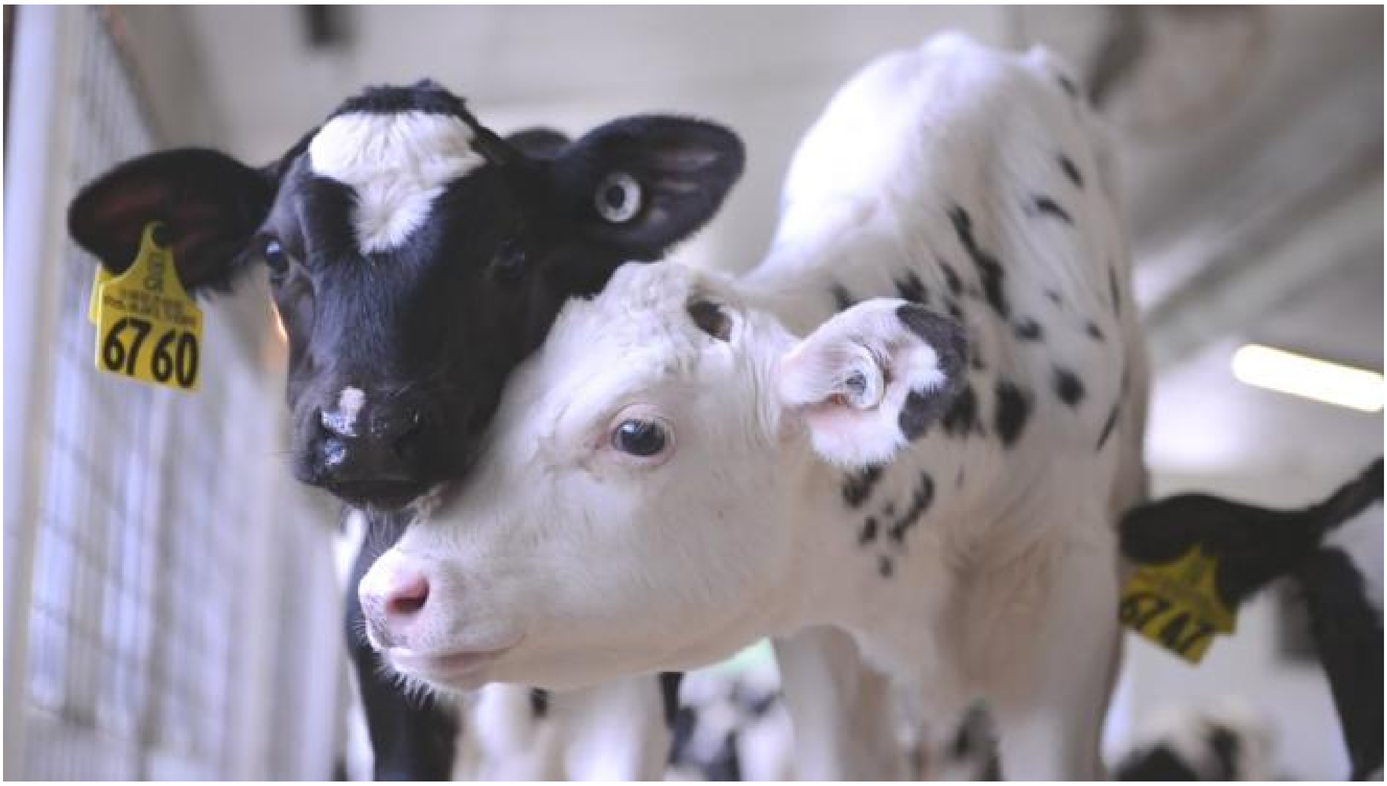

Study: Use of Apps in Veterinary Medicine – Fluid Therapy in Calves

Welcome DVM4 students!

If you consent, you will complete an online, anonymous survey that will gauge your experience using a brand-new web app that we have developed to make fluid therapy easier. In the survey you will be asked to complete two case-based exercises. For the first case, you will use your lecture notes to design a fluid therapy plan for a calf with diarrhoea. For the second case, you will complete a similar exercise using the app. After completion of the survey, you will be asked to share your thoughts on the use of apps in veterinary medicine and how the new app can be improved.

Below is a consent form with links to a participant information statement.

Please contact Sam Rowe (samuel.rowe@sydney.edu.au) if you have any questions.

In giving my consent I state that:

- I understand the purpose of the study, what I will be asked to do, and any risks/benefits involved.
- I have read the Participant Information Statement [download] and have been able to discuss my involvement in the study with the researchers (samuel.rowe@sydney.edu.au) if I wished to do so.
- The researchers have answered any questions that I had about the study and I am happy with the answers.
- I understand that being in this study is completely voluntary and I do not have to take part.
- My decision whether to be in the study will not affect my relationship with the researchers or anyone else at the University of Sydney now or in the future.

- I understand that I can withdraw from the study at any time and that incomplete surveys will be deleted.
- I understand that my questionnaire responses cannot be withdrawn once they are submitted, as they are anonymous and therefore the researchers will not be able to tell which one is mine.
- I understand that the results of this study may be published, and that publications will not contain my name or any identifiable information about me.
- I understand that a short summary of findings from this study will be circulated to DVM4 students and will not contain my name or any identifiable information about me.

[A copy of this consent declaration can be downloaded here for your records - keep this for yourself]

PLEASE INDICATE THAT YOUR CONSENT BY CLICKING THE BUTTON BELOW

- ◯ I consent to participating in this study
- ◯ I do not wish to participate

What is your age?

- ◯ [ ] Please write age in box
- ◯ Do not wish to disclose

What is your sex?

- ◯ Male
- ◯ Female
- ◯ Non-binary / third gender
- ◯ Prefer not to say

Please indicate your DVM year

- ◯ 3rd year
- ◯ 4th year

Do you currently use any apps for learning in veterinary medicine?

- ◯ Yes
- ◯ No

Please list the apps you use

Random 2 - NoApp=5d, App=3w

You will now be asked to design fluid plans for 2 scenarios. Note that there is no time limit for this.

Scenario 1

- 5 day old calf presents with profuse watery diarrhoea.
- Clinical exam finds sunken eyes (5mm), depressed mentation, absent suck reflex and unable to stand without assistance.
- Estimated bodyweight is 30kg
- Blood glucose is normal

Take as long as you need to develop a fluid plan that addresses each of the sections below. We recommend that you use your lecture notes from DVM3.

Download lecture slides from VETS6304 on fluid therapy here

What is the fluid deficit for this calf (L)?

What is the estimated ongoing losses (L / day)?

What is the estimated maintenance requirements (L / day)?

What is the estimated base deficit (aka base excess) - mmol/L or mEq/L?

Please enter your fluid plan which outlines the following details (scenario 1)

- Fluid type (may need to use more than one fluid type for a case)
- Volume required
- Rate of administration
- Additives required (eg. sodium bicarbonate)

Describe your level of confidence in the accuracy of your calculations

- ◯ Very high
- ◯ High
- ◯ Moderate
- ◯ Low
- ◯ Very low

Do you think that more practice and experience would help build confidence in calculating fluid therapy?

- ◯ Definitely yes
- ◯ Probably yes
- ◯ Might or might not

◯ Probably not

◯ Definitely not

Please attempt the next case using the web app. It can be accessed on your phone using the QR code below, or on your computer using this LINK.

Scenario 2

- 3 week old calf presents with profuse diarrhoea.
- Clinical exam finds sunken eyes (4mm), depressed mentation, absent suck reflex and inability to stand without assistance.
- Estimated bodyweight is 40kg
- Blood glucose is normal

Take as long as you need to develop a fluid plan that addresses each of the sections below. Use the app to calculate fluid requirements

What is the fluid deficit for this calf (L)?

What is the estimated ongoing losses (L / day)?

What is the estimated maintenance requirements (L / day)?

What is the estimated base deficit (aka base excess) - mmol/L or mEq/L?

Please enter your fluid plan which outlines the following details (scenario 2)

- Fluid type (may need to use more than one fluid type for a case)
- Volume required
- Rate of administration
- Additives required (eg. sodium bicarbonate)

Random 1 - NoApp=3w, App=5d

You will now be asked to design fluid plans for 2 scenarios. Note that there is no time limit for this.

Scenario 1

- 3 week old calf presents with profuse diarrhoea.
- Clinical exam finds sunken eyes (4mm), depressed mentation, absent suck reflex and inability to stand without assistance.
- Estimated bodyweight is 40kg
- Blood glucose is normal

Take as long as you need to develop a fluid plan that addresses each of the sections below. We recommend that you use your lecture notes from DVM3.

[Download lecture slides from VETS6304 on fluid therapy here]

What is the fluid deficit for this calf (L)?

What is the estimated ongoing losses (L / day)?

What is the estimated maintenance requirements (L / day)?

What is the estimated base deficit (aka base excess) - mmol/L or mEq/L?

Please enter your fluid plan which outlines the following details (scenario 1)

- Fluid type (may need to use more than one fluid type for a case)
- Volume required
- Rate of administration
- Additives required (eg. sodium bicarbonate)

Describe your level of confidence in the accuracy of your calculations

- ◯ Very high
- ◯ High
- ◯ Moderate
- ◯ Low
- ◯ Very low

Do you think that more practice and experience would help build confidence in calculating fluid therapy?

- ◯ Definitely yes
- ◯ Probably yes
- ◯ Might or might not
- ◯ Probably not
- ◯ Definitely not

Please attempt the next case using the web app. It can be accessed on your phone using the QR code below, or on your computer using this LINK.

Scenario 2

- 5 day old calf presents with profuse watery diarrhoea.
- Clinical exam finds sunken eyes (5mm), depressed mentation, absent suck reflex and unable to stand without assistance.

- Estimated bodyweight is 30kg
- Blood glucose is normal

Take as long as you need to develop a fluid plan that addresses each of the sections below. Use the app to calculate fluid requirements

What is the fluid deficit for this calf (L)?

What is the estimated ongoing losses (L / day)?

What is the estimated maintenance requirements (L / day)?

What is the estimated base deficit (aka base excess) - mmol/L or mEq/L?

Please enter your fluid plan which outlines the following details (scenario 2)

- Fluid type (may need to use more than one fluid type for a case)
- Volume required
- Rate of administration
- Additives required (eg. sodium bicarbonate)

Final questions

The next set of questions are designed to gauge your experience using the app and your attitudes towards the use of apps in veterinary medicine

If you were expected to design a fluid therapy plan for a sick calf in the field, which method would be your preference? Please answer in 10 lines or less.

How user friendly was the interface for the web app?

- ◯ Very easy to use
- ◯ Easy to use
- ◯ Neutral
- ◯ Hard to use
- ◯ Very hard to use

Do you think that a demonstration video would help to you to use the app better?

- ◯ Definitely yes
- ◯ Probably yes
- ◯ Might or might not
- ◯ Probably not
- ◯ Definitely not

Would you be more likely to use this app if it was a native mobile app (i.e. purchased from app stores and downloaded onto the phone) or a web based app?

- ◯ Native mobile app
- ◯ Web-based app
- ◯ Makes no difference to me

Do you think that veterinarians should be able to do fluid therapy on their own as a general rule without assistance from an application? Please answer this question in 10 lines or less.

Do you have any feedback on the app? How can it be improved?

Thank you! The survey is finished.

One last thing: If you are free and interested, there will be an opportunity to join a focus group that will commence in June. The objective of the focus group is to get more in-depth insights into your experience with using our fluid therapy app and to get your attitudes and opinions around the use of apps in veterinary medicine generally.

This is completely voluntary, and your identity will not be identifiable in any of the study reports / presentations. A participant information statement can be accessed here, which contains additional information about how your privacy will be protected. If you are interested, please select ‘yes’ at the end of the survey and input you email address. You will be contacted by Sam Rowe to organise a time for the focus group session.

- ◯ Yes I am interested (enter you email in the box below)
- ◯ No thanks

Use of Apps in Veterinary Medicine – Fluid Therapy in Calves

Powered by Qualtrics
